# Mutual Antagonism between Circadian Protein Period 2 and Hepatitis C Virus Replication in Hepatocytes

**DOI:** 10.1371/journal.pone.0060527

**Published:** 2013-04-08

**Authors:** Giorgia Benegiamo, Gianluigi Mazzoccoli, Francesco Cappello, Francesca Rappa, Nunzia Scibetta, Jude Oben, Azzura Greco, Roger Williams, Angelo Andriulli, Manlio Vinciguerra, Valerio Pazienza

**Affiliations:** 1 Gastroenterology Unit, IRCCS “Casa Sollievo della Sofferenza”, Hospital San Giovanni Rotondo (FG), San Giovanni, Italy; 2 Department of Medical Sciences, Division of Internal Medicine IRCCS Scientific Institute and Regional General Hospital “Casa Sollievo della Sofferenza”, San Giovanni Rotondo (FG), Italy; 3 Euro-Mediterranean Institute of Science and Technology (IEMEST), Palermo, Italy; 4 Department of Experimental Biomedicine and Clinical Neurosciences, Section of Human Anatomy, University of Palermo, Palermo, Italy; 5 Unit of Pathology, Civic Hospital, Palermo, Italy; 6 University College London (UCL)-Institute of Liver and Digestive Health, Division of Medicine, Royal Free Campus, London, United Kingdom; 7 Institute of Hepatology, Foundation for Liver Research, London, United Kingdom; St.Louis University, United States of America

## Abstract

**Background:**

Hepatitis C virus (HCV) infects approximately 3% of the world population and is the leading cause of liver disease, impacting hepatocyte metabolism, depending on virus genotype. Hepatic metabolic functions show rhythmic fluctuations with 24-h periodicity (circadian), driven by molecular clockworks ticking through translational-transcriptional feedback loops, operated by a set of genes, called clock genes, encoding circadian proteins. Disruption of biologic clocks is implicated in a variety of disorders including fatty liver disease, obesity and diabetes. The relation between HCV replication and the circadian clock is unknown.

**Methods:**

We investigated the relationship between HCV core infection and viral replication and the expression of clock genes (Rev-Erbα, Rorα, ARNTL, ARNTL2, CLOCK, PER1, PER2, PER3, CRY1 and CRY2) in two cellular models, the Huh-7 cells transiently expressing the HCV core protein genotypes 1b or 3a, and the OR6 cells stably harboring the full-length hepatitis C genotype 1b replicon, and in human liver biopsies, using qRT-PCR, immunoblotting, luciferase assays and immunohistochemistry.

**Results:**

In Huh-7 cells expressing the HCV core protein genotype 1b, but not 3a, and in OR6 cells, transcript and protein levels of PER2 and CRY2 were downregulated. Overexpression of PER2 led to a consistent decrease in HCV RNA replicating levels and restoration of altered expression pattern of a subset of interferon stimulated genes (ISGs) in OR6 cells. Furthermore, in liver biopsies from HCV genotype 1b infected patients, PER2 was markedly localized to the nucleus, consistent with an auto-inhibitory transcriptional feedback loop.

**Conclusions:**

HCV can modulate hepatic clock gene machinery, and the circadian protein PER2 counteracts viral replication. Further understanding of circadian regulation of HCV replication and rhythmic patterns of host-hosted relationship may improve the effectiveness of HCV antiviral therapy. This would extend to hepatic viral infections the current spectrum of chronotherapies, implemented to treat metabolic, immune related and neoplastic disease.

## Introduction

Basic cell functions such as proliferation, growth, differentiation, autophagy and glucose and lipid metabolism show time related fluctuations, and when the oscillations are rhythmic with a periodicity of approximately 24 h the rhythmicity is defined circadian [1]. Cellular circadian rhythmicity is driven by molecular clockworks comprised of translational-transcriptional feedback loops put in place by a set of genes, called core clock genes, coding for proteins that in turn suppress gene expression in a cycle that completes itself in one day. Clock genes are transcriptionally activated by the basic helix–loop–helix-PAS transcription factors CLOCK and ARNTL (or its paralog ARNTL2), which heterodimerize and bind to E-box enhancer elements in the promoters of the Period (*PER* 1, 2 and 3) and Cryptochrome (*CRY*1 and *2*) genes. The *PER* and *CRY* mRNAs translate into PER and CRY proteins to form a repression complex which translocates back into the nucleus, interact directly with CLOCK and ARNTL heterodimer and inhibits its transactivation [2], [3]. Notably, a growing body of evidence suggests that the feeding behavior and nutrient metabolic pathways can entrain and modulate the circadian clocks and in turn the clock gene machinery regulates multiple metabolic pathways and metabolite availability, driving the expression of clock controlled genes and transcription factors (DBP, TEF, HLF, E4BP4, DEC1-2) [4], [5], [6].

Viruses may utilize the cellular machinery to replicate, as they need host-cell replication proteins to support their own replication. Circadian variation of expression of genes that regulate the cell cycle may influence viral replication, determining daily peaks in synchrony with the cell cycle. E4BP4, a transcription factor that regulates mammalian circadian oscillatory mechanism, coordinates expression of viral genes with the cellular molecular clock and represses viral promoter sequences [7], [8]. Viral immediate-early genes appear to synchronize to 24 h rhythmicity and large DNA viruses may exhibit circadian periodicity with respect to persistent viral replication and reactivation from latency [7], [8]. Viruses are able to exploit the circadian system for optimal timing of infection and large DNA viruses show amplified DNA replication in response to terminal differentiation, suggesting a regulation mediated by circadian pathways [9].

Chronic hepatitis C virus infection (HCV) is a viral pandemic and the leading cause of liver fibrosis and cirrhosis, often progressing to liver cancer (hepatocellular carcinoma, HCC) [10]. Hepatitis C virus has evolved over a period of several thousand years and the most commonly used classification distinguishes six major genotypes. These genotypes are further divided into subtypes that differ from each other by 20–25% in nucleotide sequence, resulting in sequence diversity over the complete genome up to 35% [11]. The ability of the HCV core protein to interfere with glucose and lipid metabolic pathways and to modulate gene transcription, as well as cell proliferation and death, has been well characterized [12], [13], [14] and depends on the viral genotype: genotype 1b is the most aggressive and associated to HCC, while genotype 3a is more associated to lipid accumulation in the liver [11].

To date the interplay between HCV infection and/or replication and the clock gene machinery is unknown. To address this issue we used two *in vitro* models of HCV infection, Huh-7 cells expressing the HCV core protein of two different genotypes (1b and 3a) and OR6 cells replicating the full-length HCV genotype 1b genome, and we evaluated liver biopsies of patients with HCV infection.

## Materials and Methods

### Ethics Statement

Human biopsies: all the procedures followed were in accordance with the ethical standards of the responsible committees (institutional and national) on human experimentation and with the Helsinki Declaration of 1975 (as revised in 2008). Written informed consents were obtained from patients at the time of biopsy and the study was approved by Ethics Committee of the Civic Hospital, Palermo, Italy.

### Human Sample Collection

Formalin-fixed paraffin embedded liver biopsies were retrospectively collected from files of the Unit of Pathology of the Civic Hospital, Palermo, Italy. 5 cases were selected of HCV genotype 1b in absence of liver cirrhosis, 5 cases were also selected of HCV genotype 1b in presence of liver cirrhosis. Finally, we selected in our files 5 age-matched cases of normal liver biopsies obtained during autoptic examination of subjects without hepatic diseases. The clinical characteristics of the patients studied are summarized in Table 1, in terms of clinical history.

**Table 1:** Clinical and pathological characteristics of the patients studied.

Disease	Number ofcases	Gender(M/F)	Age range(mean)	HCV infection(genotype 1b)	HBV infection	Alcoholism
Hepatitis	5	3/2	37–73(55)	5/5	0/5	0/5
Cirrhosis	5	2/3	65–75(71)	5/5	0/5	0/5
Normal liver	5	2/3	41–70(64)	0/5	0/5	0/5

### Immunohistochemistry

Immunohistochemistry was performed by iVIEW DAB Detection Kit for Ventana BenchMark XT automated slide stainer on sections with 4–5 µm of thickness from human liver biopsies [15]. For immunostaining it has been used the primary antibody for PER2 (dilution 1∶100, Cat. No. sc-101105, Santa Cruz Biotechnology CA USA). Positive and negative controls were run concurrently. Results were semiquantitated in blind by three expert pathologists (FR, FC and NS) and percentage of positive nuclei was calculated in 10 random high power fields (at magnification of 400X).

### Cell Culture, Transfection and Serum-Shock Induced Synchronization Procedure

Human hepatoma Huh-7 cells were cultured at 37°C in 5% CO2 atmosphere in DMEM medium supplemented with 10% fetal bovine serum (FBS), 100 U/ml penicillin and 100 ng/ml streptomycin (Invitrogen Life Technologies, Milan, Italy). OR6 cells were kindly donated by Dr. Ikeda [16]. pIRES2-EGFP plasmids containing the HCV 1b core-encoding region or the 3a or GFP alone [17] and Flag-tagged pCMV Sport2 PER2 plasmid [18], were transfected into Huh-7 cells and in OR6 cells with Lipofectamine 2000 (Invitrogen Life Technologies, Milan Italy) and with Amaxa™ NucleofectorTM Kit V (Lonza, Cologne Germany), respectively, following manufacturer’s instructions. The serum shock induced synchronization was performed as follows: approximately 4×10^5^ cells/6 wells were plated the day before the experiments. At the day of the experiments, culture medium was exchanged with serum-rich medium (DMEM containing 50% FBS) and after 2 hours this medium was replaced with serum free DMEM. The cells were harvested at different time points: 1 h, 4 h, 10 h, 16 h, 22 h and 28 h after serum shock.

### Luciferase Assays

Luciferase assays to monitor HCV replication in OR6 cells were performed as previously described [16], using a luciferase reporter assay (Promega, Madison, WI, USA).

### Quantitative Real Time PCR

Total RNA was extracted from HCV-core transfected Huh-7 cells and from OR6 cells replicating the full length HCV genome using the RNeasy® Mini Kit (Qiagen S.p.a. Milan, Italy) and subsequently digested by DNase I. cDNA was synthesized from 100 ng total RNA with Quantifast RT-PCR kit (Qiagen). For real-time PCR, we used the following SYBR Green QuantiTect Primers purchased from Qiagen: RevErbα (QT00000413), RoRα (QT00072380), ARNTL (QT00068250), ARNTL2 (QT00011844), CLOCK (QT00054481), PER1 (QT00069265), PER2 (QT00011207), PER3 (QT00097713), CRY1 (QT00025067) and CRY2 (QT00094920). For HCV quantification the following primers were used: Forward 104 (5′-AGA GCC ATA GTG GTC TGC GG-3′) and Reverse 197R (5′-CTT TCG CGA CCC AAC ACT AC-3′) described in [33]. Reactions were set up in 96-well plates using a 7700 Real-Time PCR System (Applied Biosystems, Foster City, CA, USA) and all samples were assayed in triplicate. Expression levels of target gene were normalized using the housekeeping control gene TATA binding protein (TBP, QT00000721).

### Western Blotting

Huh-7 control cells and cells transfected with either genotype HCV core protein 1b, HCV core 3a or with the empty vector were lysed and processed for immunoblotting analysis with specific antibodies as previously described [19]. Quantitative measurements of bands were performed using the NIH-Image analysis program Scion IMAGE (Scion Corp., Frederick, MD, USA).

### Antibodies

Rabbit and mouse polyclonal antibody directed against ARNTL2, ClOCK, PER1, PER2, CRY1, CRY2 and β-actin were from Santa Cruz Biotechnology, Santa Cruz, CA, USA. ARNTL and Rev-Erbα antibodies were purchased from Millipore S.p.a., Milan, Italy. The monoclonal anti-core (C7–50) antibody was obtained from Vinci-Biochem (Florence, Italy).

### Statistical Analysis

Results are expressed as means ± SE of at least three different experiments. Comparisons were made using Student’s t-test as appropriate. Differences were considered as significant at P<0.05.

## Results

### Altered Clock Genes Expression in OR6 Replicating the Full Length HCV RNA

As viruses are highly dependent on cellular machinery for replication, it was proposed that the viral replication may be synchronized to the molecular clockwork and that in turn the circadian clock may influence viral replication [7].

**Figure 1:**
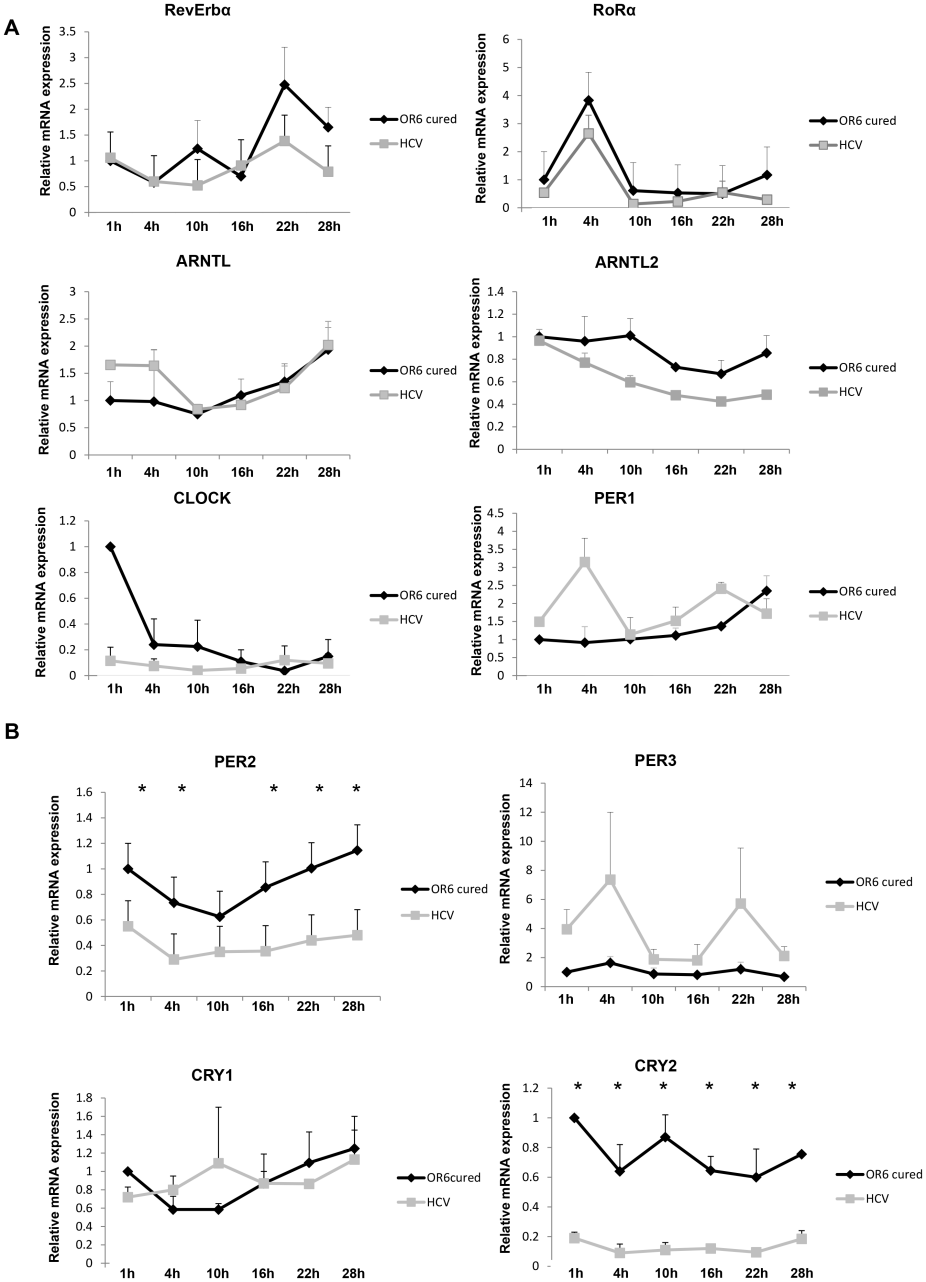
qRT-PCR analysis of clock gene mRNA expression in OR6 control cells (cured, not expressing the HCV 1b full replicon) and in HCV replicating OR6 cells (HCV). Control (cured) OR6 cells and OR6 cells replicating HCV genotype 1b were serum shocked and RNA was extracted every 1 h, 4 h, 10 h, 16 h, 22 h and 28 h after serum shock. mRNA levels of Rev-Erbα, Rorα, ARNTL, ARNTL2, CLOCK, PER1, PER2, PER3, CRY1 and CRY2 genes were assessed by qRT-PCR (**A** and **B**). Values were normalized against TBP as housekeeping control gene. Results are expressed as means ± SE of three independent experiments. * = p<0.05 in HCV replicating cells versus control cured OR6 cells.

**Figure 2:**
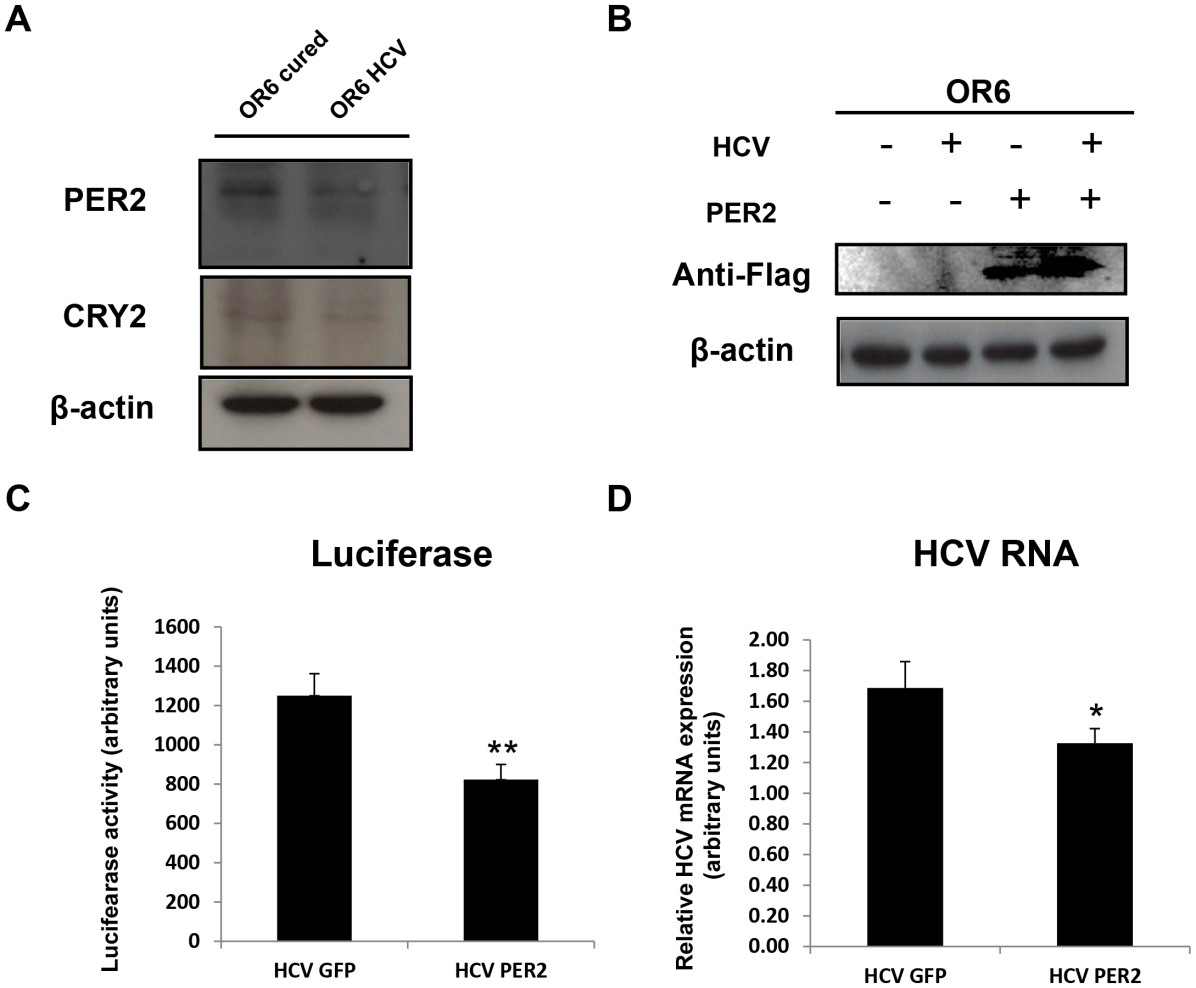
Mutual antagonism between HCV genotype 1b replication and PER2 expression in OR6 cells. (**A**) Representative immunoblots of PER2 and CRY2 protein expression in HCV replicating OR6 cells versus control (cured) cells. 1 out of 3 blots is showed. β-actin was used as a loading control. (**B**) PER2 overexpression in HCV replicating OR6 cells using a Flag-tagged construct. HCV replicating OR6 cells or control OR6 cells were electroporated with Flag-PER2, 24 hours after electroporation protein lysates were processed for immunoblotting and Flag levels were detected with a specific antibody. β-actin was used as a loading control. (**C**) HCV replication was detected by a luciferase reporter assay in HCV replicating OR6 cells 24 hours post electroporation with a control (GFP) or Flag-PER2 construct. (**D**) qRT-PCR to detect HCV RNA levels upon control (GFP) or Flag-PER2 plasmid electroporation in HCV replicating OR6 cells HCV RNA values were normalized against TBP as housekeeping control gene (**C** and **D**). Results are expressed as means ± SE of three independent experiments. * = p<0.05, ** = p<0.01 in HCV replicating OR6 cells overexpressing Flag-PER2 versus HCV replicating OR6 cells overexpressing GFP.

With this premise in mind, we sought to analyze by qRT-PCR the time-qualified expression of a panel of clock genes (Rev-Erbα, Rorα, ARNTL, ARNTL2, CLOCK, PER1, PER2, PER3, CRY1 and CRY2) in OR6 cells, which express full length HCV replicon of genotype 1b [16]. The latter was able to perturb clock gene expression as shown in Figure 1A and B. A significant downregulation of CRY2 mRNA levels was observed over all the six time points, while PER2 mRNA was significantly decreased at 1 h, 10 h, 16 h, 22 h and 28 h after serum shock. Rev-Erbα, Rorα, ARNTL mRNA levels did not show statistically significant changes between OR6 cells lacking a functional HCV 1b full replicon (hereafter referred to as “cured”) and HCV-infected OR6 cells (Fig. 1A). CLOCK mRNA resulted significantly downregulated at 1 h after serum shock in OR6 induced to express HCV full length RNA when compared to cured OR6 cells (Fig. 1A). ARNTL2 mRNA levels showed a trend, though not reaching statistical significance, towards a decrease over all the time points considered in HCV-infected compared to cured OR6. Moreover, time related patterns of expression of PER1 and PER3 were asynchronous in induced OR6 as compared to control cells. We then sought to confirm if PER2 and CRY2 mRNA dowregulation was similarly observed at the protein level. PER2 and CRY2 proteins were found decreased in OR6 HCV replicating cells as compared to control cells (Figure 2A).

### PER2 Overexpression Hampers HCV RNA Replication

In order to elucidate the interplay between the clock gene machinery and HCV replication, we decided to focus our attention on the role of PER2, as its role in regulating the daily rhythm of IFN-γ and its tumor suppressor activity have been already demonstrated [20], [21]. For this purpose, we overexpressed Flag-tagged Per2 protein [18] in OR6 cells replicating the HCV genotype 1b full length RNA (Figure 2B). The efficiency of transfection was about 50–60% in OR6 cells (*data not shown*). As previously described, OR6 cells contain a very efficient luciferase reporter system for monitoring HCV RNA levels [16]. Upon PER2 overexpression, we observed approximately 35% reduction in luciferase activity in HCV-expressing OR6 cells compared to untransfected cells (Fig. 2C). Consistently, HCV RNA levels were significantly reduced by 27% in PER2-overexpressing OR6 cells, as assessed by qRT-PCR (Fig. 2D). Altogether, these data demonstrate for the first time that circadian protein PER2 can hinder the replication of HCV genotype 1b.

### Interferon Stimulated Genes in OR6 Cells Overexpressing PER2 Protein

Biomolecules mediating innate immune defenses, such as the Interferon Stimulated Genes (ISGs) products, can prevent the translation of HCV and cellular mRNAs to limit viral replication and can also initiate apoptosis if the cell is overwhelmed. In order to replicate, HCV machinery can interact directly with ISGs and neutralize their expression and function. To understand the role of PER2 in diminishing HCV RNA replication we evaluated by qRT-PCR the mRNA expression levels of a subset of ISGs (OAS1, Mx1, IRF9, PKR) in PER2 overexpressing OR6 HCV RNA replicating and cured cells as compared to GFP-transfected OR6 HCV replicating and cured cells.

**Figure 3:**
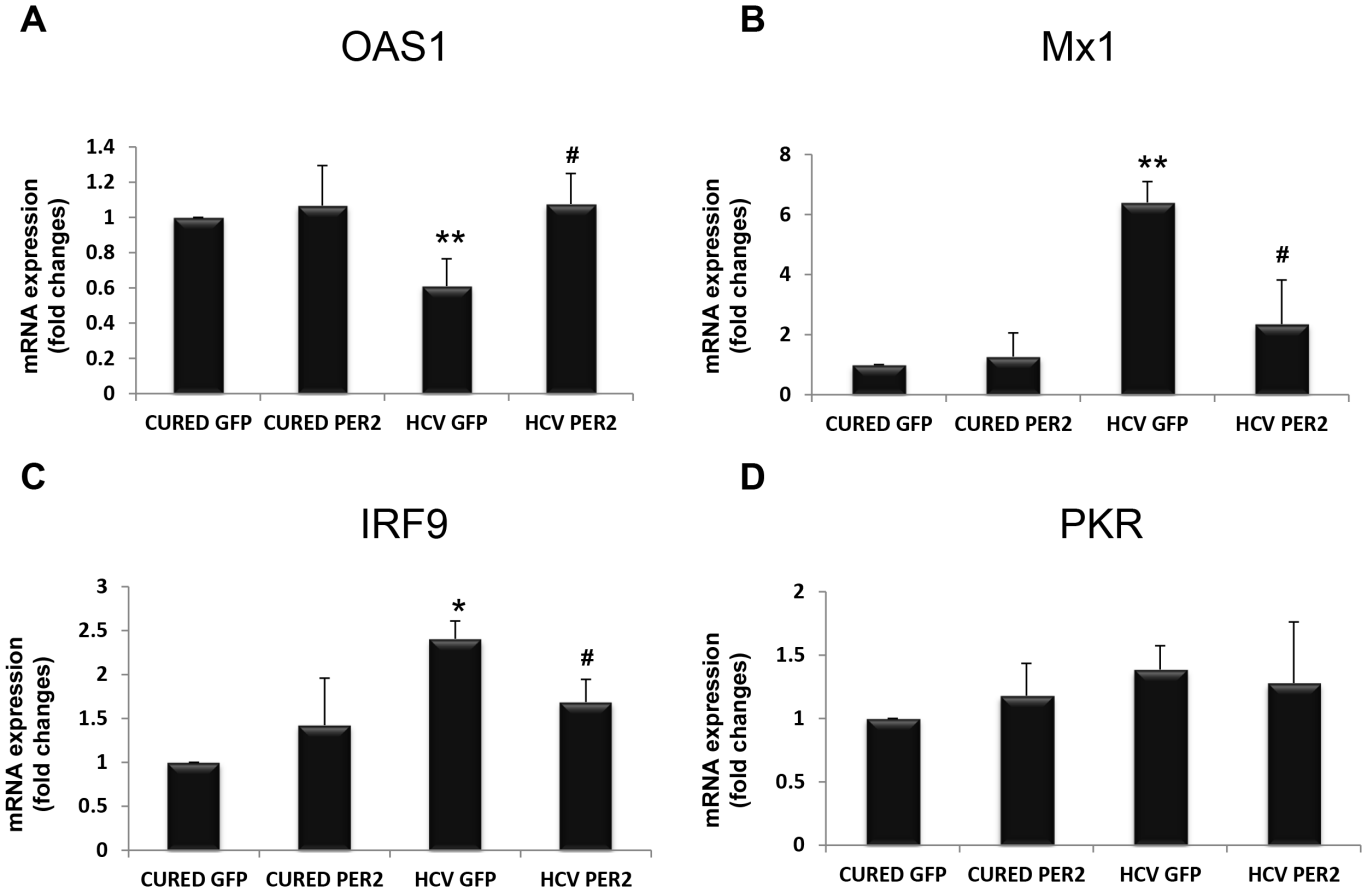
qRT-PCR analysis of interferon stimulated genes (ISGs: OAS1, Mx1, IRF9, PKR) mRNAs expression levels in control (cured) OR6 cells and HCV replicating OR6 cells electroporated with GFP (cured GFP, HCV GFP) or with Flag-PER2 (cured PER2, HCV PER2) A, B, C and D. mRNA was extracted 24 hours after electroporation with the respective constructs and ISGs levels were normalized to TBP as housekeeping gene. Results are expressed as means ± SE of three independent experiments. * = p<0.05, ** = p<0.01 in HCV replicating OR6 cells overexpressing GFP versus OR6 control cells overexpressing GFP or Flag-PER2. # = p<0.05 in HCV replicating OR6 cells overexpressing Flag-PER2 versus HCV replicating OR6 cells overexpressing GFP.

OR6 cells expressed OAS1, Mx1, IRF9 and PKR at the mRNA level, both in cured and infected cells (Figure 3, A-D). PER2 overexpression had no effect in cured cells, compared to the condition of GFP overexpression. OAS1 mRNA levels were significantly decreased, and Mx1 and IRF9 were significantly increased exclusively in GFP-transfected OR6 HCV replicating cells (Figure 3, A–D). In turn, PER2 overexpression was able to efficiently restore OAS1, whilst Mx1 and IRF9 mRNA levels returned to the basal level in OR6 HCV replicating cells. PKR expression did not display significant changes in any of the conditions tested (Figure 3, A–D).

### Clock Gene Expression in Huh-7 Cells Expressing the HCV Core Protein of Genotype 1b or 3a

**Figure 4:**
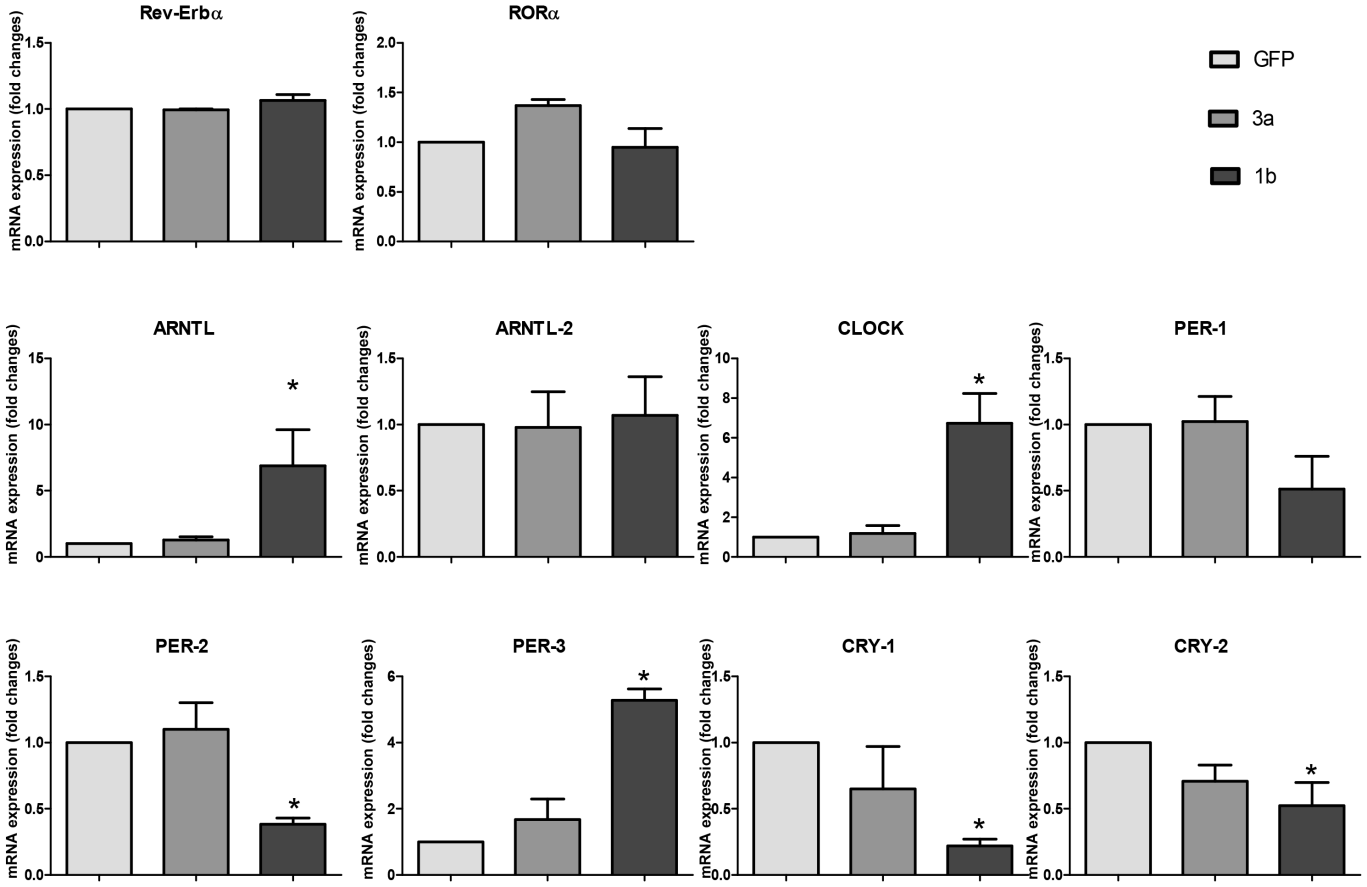
qRT-PCR analysis of clock gene mRNAs expression levels in Huh-7 cells overexpressing the HCV core proteins genotype 1b and 3a. Huh-7 cells were transiently transfected with HCV core proteins 1b and 3a as previously described [17], or with GFP. 48 hours after transfection mRNA levels of Rev-Erbα, Rorα, ARNTL, ARNTL2, CLOCK, PER1, PER2, PER3, CRY1 and CRY2 genes were assessed by qRT-PCR. Values were normalized against TBP as housekeeping control gene. Light gray: GFP transfected cells; dark gray: HCV core protein genotype 3a transfected cells; black: HCV core protein genotype 1b transfected cells. Results are expressed as means ± SE of three independent experiments. * = p<0.05 in HCV core proteins transfected versus GFP transfected control cells.

To investigate a possible role of HCV core proteins in the impairment of the clock gene machinery, we evaluated by qRT-PCR the mRNA expression levels of a panel of clock genes (Rev-Erbα, Rorα, ARNTL, ARNTL2, CLOCK, PER1, PER2, PER3, CRY1 and CRY2) in an additional relevant cell line, human hepatoma Huh-7 cells transiently expressing the HCV core protein of genotype 1b or 3a [17]. As shown in Figure 4, ARNTL, CLOCK and PER3 mRNA levels resulted significantly increased by the HCV core protein 1b, while PER2, CRY1 and CRY2 were found significantly decreased. These results were consistent with those obtained in the OR6 cell line (Fig. 1, A and B). No alterations were observed in Huh-7 cells transfected with HCV core protein of genotype 3a (Fig. 4). Rev-Erbα, Rorα, and ARNTL2 mRNA levels were not changed in either of the genotypes. In regard to PER1 expression, HCV core protein genotype 1b tended to induce decrease in its mRNA expression level, without reaching statistical significance.

### Immunoblot Detection of Circadian Proteins in Huh-7 Cells Expressing the HCV Core Protein of Genotype 1b or 3a

**Figure 5:**
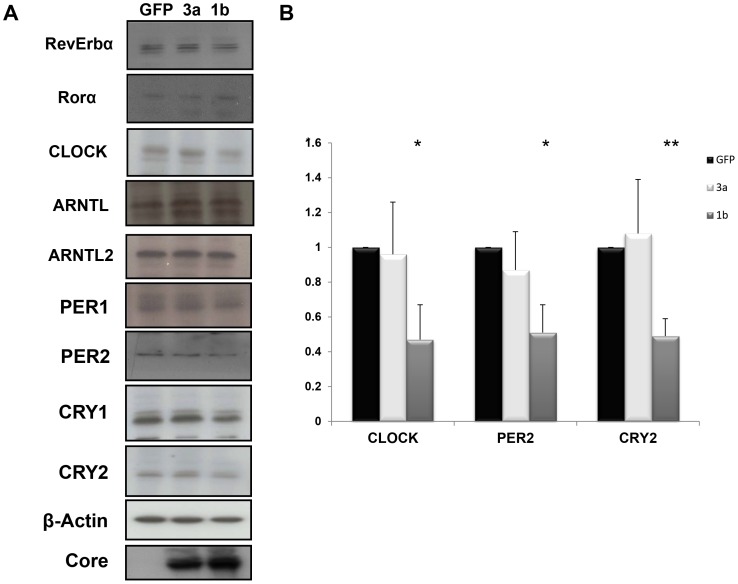
Immunoblot detection of circadian proteins in Huh-7 cells expressing the HCV core protein genotype 1b or 3a and GFP-expressing control cells. (**A**) 48 hours after transfection cells were lysed and equal amounts of proteins were loaded on a 10% polyacrylamide gel, separated by electrophoresis and immunoblotted with specific Rev-Erbα, Rorα, CLOCK, ARNTL, ARNTL2, PER1, PER2, CRY1 and CRY2 primary antibodies. β-actin expression served as loading control. (**B**) Densitometric quantification of CRY2, PER2 and CLOCK proteins normalized to β-actin expression of three different experiments.

To corroborate the mRNAs expression profile data and with the intent to assess whether the dysregulation of clock gene expression levels resulted in a differential expression pattern at protein level, we analyzed Rev-Erbα, Rorα, ARNTL, ARNTL2, CLOCK, PER1, PER2, CRY1 and CRY2 by immunoblotting 48 hours after HCV core transfection in Huh-7 cells. As shown in Figure 5, HCV core protein of genotype 1b was able to decrease CLOCK, PER2, CRY1 and CRY2 protein levels. Rev-Erbα, Rorα, ARNTL, ARNTL2 and PER1 protein levels were unmodified. In regard to the HCV core genotype 3a, only CLOCK protein expression resulted increased at the same time point and no other changes were observed (Figure 5, A and B). The differences between CLOCK mRNA and protein levels in both conditions of HCV core protein 1b and 3a overexpression are suggestive of a post-transcriptional mechanism of regulation of CLOCK protein in Huh-7 cells. Post-transcriptional regulations are crucial for the rhythmic activity of the circadian molecular clockworks, and in particular have been shown to play a part in the generation of time related variations of CLOCK protein levels in the Drosophila Melanogaster [22].

### PER2 in the Liver of Patients Infected with HCV Genotype 1b

**Figure 6:**
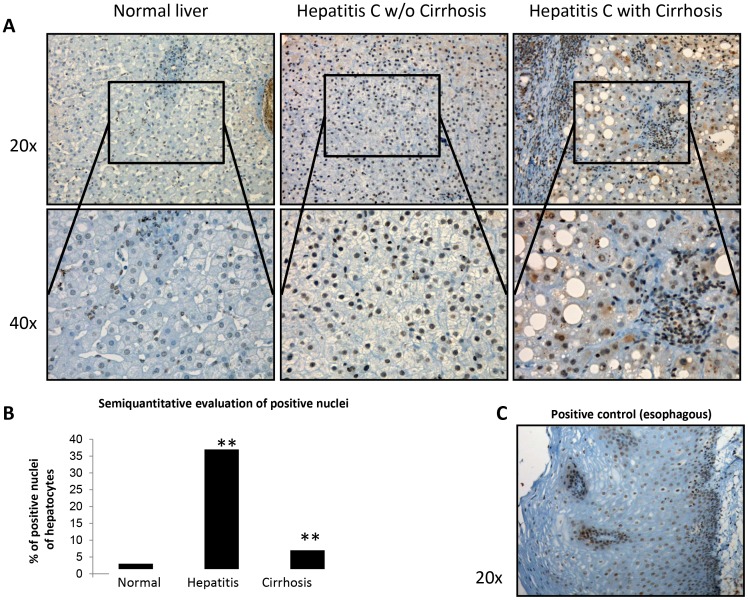
Representative panel of immunostainings performed for PER2 in samples of normal liver, hepatitis related to HCV (genotype 1b positive) and cirrhosis arisen on HCV infection (genotype 1b positive) (**A**)**.** Positivity of nuclei of hepatocytes in hepatitis was significantly higher compared with those in cirrhosis. The latter was comparable to that of hepatocytes in normal liver. In cirrhosis, PER2 Immunopositivity is also observed in the cytoplasm of hepatocytes. Positive control of esophagus showed immunopositivity in the nuclei of all cells (**C**). Pictures showed the same area observed with a lower (above) and higher (below) magnification. (**B**) Histogram shows statistical results for the evaluation of immunopositivity for PER2. Significant differences (** = p<0.005) in the percentage of positive nuclei were found between biopsies of hepatitis and either cirrhosis and normal liver.

We next sought to confirm if the downregulation of PER2 protein observed in the two *in vitro* HCV genotype 1b cell models (Huh-7 and OR6) was found in the patho-physiological context of liver of patients infected with HCV genotype 1b (whose clinical characteristics are reported in Table 1). Immunostaining for PER2 showed significant differences (p<0.005) in the percentage of positive nuclei between hepatitis and, either, cirrhosis or normal liver. In particular, 37% of nuclei of hepatocytes in hepatitis showed immunopositivity for PER2, while only 7% of nuclei of hepatocytes in cirrhosis and 3% of nuclei of hepatocytes in normal liver were immunopositive. In cirrhosis, PER2 Immunopositivity was also observed in the cytoplasm of hepatocytes (Fig. 6).

## Discussion

Both HCV infection and disruption of the cellular circadian clock have been shown to heavily impact on hepatic lipid and glucose metabolism inducing enhanced lipid accumulation, condition that can predispose to serious liver disorders such as steatohepatitis, cirrhosis and hepatocellular carcinoma (HCC) [6], [10]. Clock genes regulate the timing of DNA repair, apoptosis, and cell proliferation, processes that when altered are hallmarks of carcinogenesis. About 5% to 15% of genome-wide mRNA expression exhibits a circadian rhythm of oscillation driven by the clock genes [23], [24], including some established tumor suppressor genes and oncogenes [23], [24]. Mutation or dysregulation of clock genes have been associated with increased susceptibility to HCC and other cancer types in several animal models as well as in humans [25], [26], [27].

To our knowledge, the interactions between the cellular circadian clock and HCV life cycle in hepatocytes have not been studied up until now. We show here for the first time that HCV genotype 1b, but not 3a, induced profound alterations in the mRNA and protein expression of the clock gene machinery in two cellular models, the OR6 cells harboring the full replicon of HCV genotype 1b and in Huh-7 cells expressing HCV core protein. Consistent findings indicated downregulation of PER2 and CRY2 proteins by HCV genotype 1b in both cellular models. Interestingly, downregulation of PER2 and CRY2 occurs also in fibrotic livers and HCC [27], [28]. In addition, high expression of *PER2* gene was associated with significantly better outcomes in liver metastasis of colorectal carcinoma [29], and high PER2 protein levels are protective from carbon tetrachloride-induced hepatotoxicity [30].

**Figure 7:**
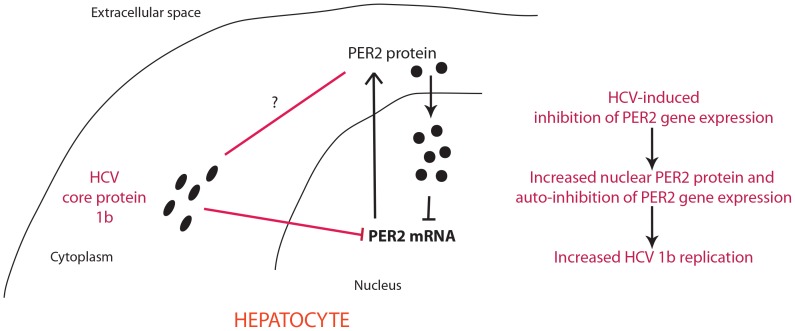
Scheme illustrating the antagonism between HCV genotype 1b replication and PER2 in hepatocytes. HCV core protein genotype 1b, via a yet undefined mechanism, induces a downregulation of PER2 mRNA. PER2 protein produced in the cytoplasm accumulates in the nucleus, as observed in human liver biopsies infected with HCV genotype 1b, where could further inhibit the production of its own RNA [2], [3]. Alteration of this equilibrium by exogenous overexpression of PER2 protein hampers HCV genotype 1b replication.

In this study, we focused on the role of PER2 on HCV replication, as this circadian protein regulates the rhythms of IFN-γ signaling, critical for innate and adaptive immunity against HCV infection [20], [21], while CRY2 is involved in NF-kB activation and pro-inflammatory processes [31]. A recent elegant study showed that PER2, rather than CRY proteins, is the critical nodal point for circadian oscillations in cells and in the intact organism [32]. Our analysis of human liver biopsies revealed that HCV genotype 1b induces a massive increase in nuclear PER2 nuclear localization in hepatocytes in hepatitis in absence of cirrhosis compared to control livers, while PER2 immunopositivity is also observed in the cytoplasm of HCV genotype 1b infected hepatocytes. Interestingly, in chronically infected HCV patients, as well as in cirrhosis, hepatocytes at late stage of infection dominate the cell population, resulting in the production of high density, poorly infectious HCV particles, while in acutely infected non cirrhotic patients there is a production of low density highly infectious HCV particles [33]. We speculate that this may have an effect on PER2 subcellular localization: an increase in nuclear localization together with a decrease in the mRNA and total protein levels in cell models is consistent with the fact that nuclear PER2 forms a repression complex that interacts directly with the core clock machinery blocking its own production [2], [3] (Figure 7). Nevertheless, further in depth *in vivo* and *in vitro* studies are required to understand the mechanisms of PER2 intracellular trafficking and degradation dependent on HCV infection.

One of the most remarkable findings was that exogenous overexpression of PER2 protein in OR6 cells hampered HCV RNA replication, which was found to be decreased by ∼30%. Consistently, PER2 overexpression influenced the HCV-dependent altered expression of ISG products, OAS1, Mx1, IRF9, which prevent the translation of HCV and cellular mRNAs to limit viral replication [34]. One of the major antiviral mechanisms of interferon is the activation of OAS1, which leads to the production of short oligonucleotides that in the presence of viral infections activate ribonucleases that destroy viral mRNA within infected cells inhibiting viral replication [35]. Noteworthy, in our study Per2 overexpression potentiated the activation of OAS1, suggesting a possible mechanism involved into the observed reduction of HCV RNA levels. Although exogenous PER2 overexpression reduced the apparently coherent elevated levels of Mx1 and IRF9 in OR6 replicating the HCV RNA, we assume that some viral components act to modulate viral load by means of the activation or inhibition of host defense proteins in order to maintain low steady levels of virus in the infected cells, enabling HCV to escape from the host immune surveillance, and facilitating persistent viral infection.

In conclusion, we found that HCV core protein genotype 1b is able to impair the clock gene machinery suggesting that HCV may adopt this strategy to better exploit the host-cell replication machinery. On the other hand, overexpression of the circadian protein PER2 hampers HCV RNA replication. Circadian regulation of viral replication has potential applications in the development of therapeutic strategies. Circadian rhythm-based treatments (i.e. chronotherapies), have been employed against metabolic, immune-related and neoplastic diseases [36], [37]. Standard therapy for HCV patients involves administration of interferon-α and ribavirin (a nucleoside analogue) [11], [34]. Recently, an interferon/ribavirin-free therapy based on newly identified and efficacious protease inhibitors (telaprevir, boceprevir) promisingly entered into the clinic to treat HCV patients [38].

In light of our findings, the new strategies to inhibit viral replication could address the circadian relationship between host cell and hosted viruses, with the aim to improve the efficacy of treatment modalities through optimized timing of therapeutic regimens, minimizing the toxicity of pharmacological agents and targeting in a better way virus replication.
